# The microRNA target site profile is a novel biomarker in the immunotherapy response

**DOI:** 10.3389/fonc.2023.1225221

**Published:** 2023-12-21

**Authors:** Yulong Bai, Yujia Li, Yidi Qin, Xinshuo Yang, George C. Tseng, Soyeon Kim, Hyun Jung Park

**Affiliations:** ^1^ Department of Human Genetics, University of Pittsburgh, Pittsburgh, PA, United States; ^2^ Statistics-Oncology, Eli Lilly and Company, Indianapolis, IN, United States; ^3^ Department of Operations Research and Financial Engineering, Princeton University, Princeton, NJ, United States; ^4^ Department of Biostatistics, University of Pittsburgh, Pittsburgh, PA, United States; ^5^ Department of Pediatrics, School of Medicine, University of Pittsburgh, Pittsburgh, PA, United States

**Keywords:** RNA-level regulation, microRNA binding mechanism, tumor initiation, cancer biology, biomarker

## Abstract

MicroRNAs (miRNAs) bind on the 3′ untranslated region (3′UTR) of messenger RNAs (mRNAs) and regulate mRNA expression in physiological and pathological conditions, including cancer. Thus, studies have identified miRNAs as potential biomarkers by correlating the miRNA expression with the expression of important mRNAs and/or clinical outcomes in cancers. However, tumors undergo pervasive 3′UTR shortening/lengthening events through alternative polyadenylation (APA), which varies the number of miRNA target sites in mRNA, raising the number of miRNA target sites (numTS) as another important regulatory axis of the miRNA binding effects. In this study, we developed the first statistical method, BIOMATA-APA, to identify predictive miRNAs based on numTS features. Running BIOMATA-APA on The Cancer Genome Atlas (TCGA) and independent cohort data both with immunotherapy and no immunotherapy, we demonstrated for the first time that the numTS feature 1) distinguishes different cancer types, 2) predicts tumor proliferation and immune infiltration status, 3) explains more variation in the proportion of tumor-infiltrating immune cells, 4) predicts response to immune checkpoint blockade (ICB) therapy, and 5) adds prognostic power beyond clinical and miRNA expression. To the best of our knowledge, this is the first pan-cancer study to systematically demonstrate numTS as a novel type of biomarker representing the miRNA binding effects underlying tumorigenesis and pave the way to incorporate miRNA target sites for miRNA biomarker identification. Another advantage of examining the miRNA binding effect using numTS is that it requires only RNA-Seq data, not miRNAs, thus resulting in high power in the miRNA biomarker identification.

## Introduction

MicroRNAs (miRNAs) are non-coding RNAs that bind their target sequences in messenger RNAs (mRNAs), known as miRNA response elements (MREs). Although some miRNAs can bind to the 5′ untranslated region (1–3) (5′ UTR) or open reading frames (4–6) of mRNAs, it has been a general consensus that miRNAs mainly target 3′UTRs of mRNA, as a functional MRE in 3′UTR loses its ability to mediate miRNA binding affinity when moved to the protein coding sequence (CDS) (7). During tumor initiation and progression, molecular mechanisms, including the miRNA-mediated mRNA binding mechanism on the 3′UTRs, evolve into a neoplastic state characterized by cancer hallmarks. Thus, studies have identified miRNAs as potential biomarkers for various cancer hallmarks by correlating the miRNA expression with the expression of important mRNAs and/or clinical outcomes (8, 9). Despite substantial progress in this direction, miRNA biomarkers still suffer from poor reproducibility and thus have difficulty translating (10). The poor reproducibility is mainly attributable to the failure to consider another important regulatory axis, the miRNA target sites on mRNAs. The miRNA target sites play important roles in determining the binding effect, as the binding can be viewed as a stoichiometry process between miRNA molecules and the target sites (11, 12). For example, by conducting experiments on the miRNA-induced silencing complexes (miRISCs), Mayya and Duchaine experimentally identified miRNA target sites that help explain the binding effect of particular mRNAs, among several parameters (13).

**Figure 1:**
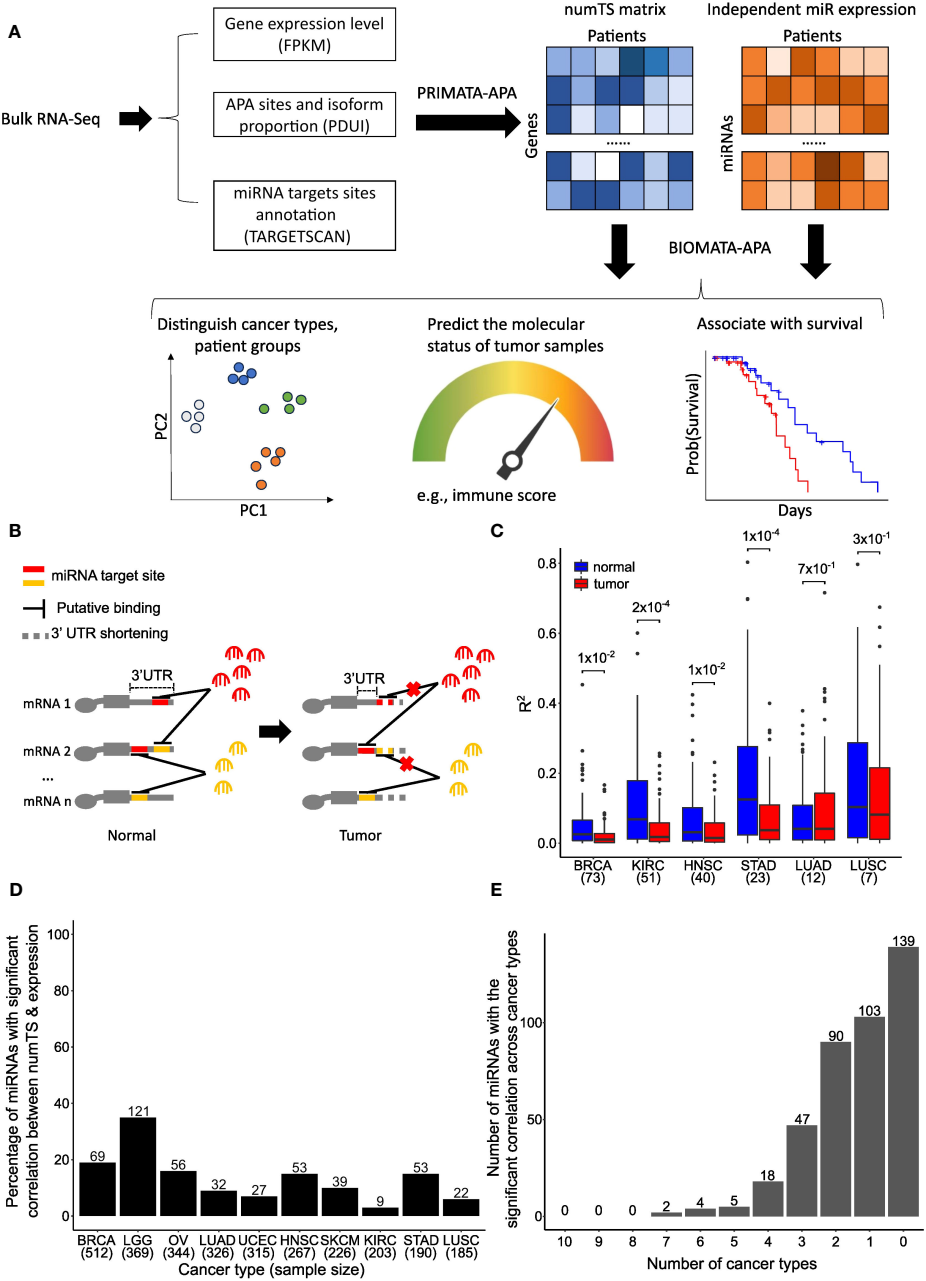
Number of miRNA target sites (numTS) is a novel biomarker independent of miRNA expression. **(A)** Representative figure showing the BIOMATA-APA steps and application scenarios. **(B)** Regardless of miRNA abundance, 3′UTR in tumors causes the loss of miRNA target sites and disrupts the competitive binding activity of different types of miRNAs. **(C)** The squared correlations between miRNA expression and numTS in normal samples are higher than in tumor samples. Wilcoxon test p-values are indicated above boxes. **(D)** Percentage of miRNAs with significant correlation between miRNA expression and numTS. The number above each bar indicates the exact number of miRNAs. **(E)** Overlap of miRNAs with the significant correlation between miRNA expression and numTS across cancer types. miRNA, microRNA. Created with Biorender.com.

Explicit modeling of miRNA target sites is even more critical to studying cancer mechanisms and cancer patients’ responses to immunotherapy. Tumors undergo pervasive 3′UTR shortening/lengthening events through alternative polyadenylation (APA) (14–16). APA can produce mRNA transcripts of varying 3′UTR lengths derived for the same genes by polyadenylating on one of the multiple polyadenylation sites (polyA sites) (17). Since miRNA target sites are enriched in the 3′UTR of mRNAs, APA consequently varies the number of miRNA target sites in mRNA (**Figure 1B**). Previously, we demonstrated that APA drives tumorigenesis by redirecting microRNA bindings to repress tumor suppressors (18). In addition, APA-mediated miRNA binding site modification may also play an important role in eliciting responses to immune checkpoint blockade (ICB) therapy. Previously, we reported that the global APA events collectively modify the binding sites of the miRNAs that not only are enriched for cancer development and treatments but also indicate immune cell infiltration to the tumor microenvironment, which is an important indicator to predict patients’ response to ICB therapy (19). Specifically, miRNA binding mechanisms are involved in inducing immune response in cancer patients by recruiting and activating immune cells within the tumor microenvironment and targeting particular cancer-related pathways in the immune cells, leading to the secretion of immunosuppressive or immunostimulating factors by either cancer cells or immune cells (20). However, existing approaches potentially lead to an incorrect estimation of the miRNA binding effect in such cases since they identify miRNA biomarkers based mostly on the expression without considering the number of miRNA target sites (21, 22). For example, a miRNA expression-based approach could identify some miRNAs as biomarkers by correlating the expression levels with immune cell infiltration in the tumor microenvironment. However, mRNAs important for immune cells may shorten the 3′UTRs and thus cannot be regulated by the miRNAs. Then, some of the highly expressed miRNAs may not play a role in the infiltration, questioning the validity of the expression-based miRNA biomarker identification.

Based on this novel concept, we propose the number of miRNA target sites (numTS) as an important biomarker in cancer. NumTS was first identified in breast tumor samples in our previous work using a computational framework and probabilistic inference of microRNA target site modification through APA (PRIMATA-APA). Although the actual miRNA binding mechanism is determined by multiple parameters (13), PRIMATA-APA analysis demonstrated that the large-scale behavior of the miRNA binding effect can be largely estimated by two main factors, miRNA expression information and the corresponding numTS values (18, 19). By evaluating the large-scale miRNA binding effect changes from normal to tumor, PRIMATA-APA showed that the pervasive APA events modify the binding effect of the miRNAs enriched for cancer development and treatments. However, PRIMATA-APA has several limitations to systematically identify miRNA numTS features as a biomarker. First, it requires both tumor and matched normal samples. Since many cancer types either do not provide matched normal samples or provide a smaller set of normal samples, this limitation reduces statistical power and the number of cancer types to analyze. Second, due to the high dimensionality of the data compared to small numbers of available samples (often referred to as the p >> n problem), the previous PRIMATA-APA association analysis suffered from a large number of uninformative or highly correlated numTS features, making it challenging to build a predictive model. Third, the previous model does not directly demonstrate the prognostic value of numTS, which is important to show clinical applicability.

To address these limitations and expand the analysis to a broader range of cancer types, we developed the first statistical method, a biomarker of microRNA target site modification through APA (BIOMATA-APA), to train predictive signatures based on numTS features (**Figure 1A**). First, by allowing a multi-direction comparison of the miRNA binding effect in the equation (see Materials and Methods), BIOMATA-APA can estimate the binding effect in tumor samples of different cancer types as well as tumor *vs.* normal samples. Second, using bootstrapping, BIOMATA-APA can select stable numTS features that constantly predict response through and filter out uninformative and highly correlated features. Finally, we evaluated the numTS features’ potential prognostic value in addition to common clinical variables using the Cox proportional-hazards model and least absolute shrinkage and selection operator (LASSO) regression. Collectively, by running BIOMATA-APA on 10 cancer types in The Cancer Genome Atlas (TCGA) and independent cohort data both with immunotherapy and no immunotherapy, we demonstrated that the numTS-based predictive signatures outperform the miRNA expression features in differentiating cancer types and predicting tumor proliferation, immune infiltration status, and abundance score of six immune cell types. Also, BIOMATA-APA revealed that the numTS features of specific miRNAs enhance the prognostic power of clinical features, while the expression of these miRNAs does not have the same advantage. Altogether, we proposed a statistical method BIOMATA-APA to reveal that the dynamics of miRNA target sites could be a better and more effective biomarker than miRNA expression to reflect the miRNA-mediated regulation underlying tumor progression and tumor response to immunotherapies.

Another advantage of BIOMATA-APA and numTS is that it allows us to estimate the miRNA binding effect using RNA-Seq data without having to sequence miRNAs since numTS features are estimated from the RNA-Seq data. Since miRNA sequencing (miRNA-Seq) is often considered an extra step to regular RNA sequencing, RNA-Seq data are more available, usually with a larger sample size than miRNA-Seq data. Thus, BIOMATA-APA enables us to analyze the miRNA binding activity with a larger sample size than with miRNA expression, resulting in higher power. Indeed, this advantage enables us to demonstrate the reproducibility of numTS features in another independent LUAD cohort [Seo et al. (23)] and to represent the treatment effect of ICB therapy, showing a separation between responder post-treatment samples and responder pre-treatment samples, although the cohorts did not have the miRNA expression sequencing data.

## Materials and methods

### Datasets

Gene expression matrices, survival information, and clinical features were downloaded from The Cancer Genome Atlas data portal (GDC portal, https://portal.gdc.cancer.gov). MiRNA expression matrices were downloaded from UCSC Cancer Genomics Hub (Xena Browser, https://xenabrowser.net/hub/). The miRNA family with aggregated (by miRNA family) average expression ≥0.01 TPM was kept in the analyses. The APA estimations, percentage of distal polyA site usage index (PDUI) (15), of TCGA tumor samples were downloaded from The Cancer 3′UTR Atlas (TC3A, http://tc3a.org/) (24). The APA estimations of TCGA tumor and normal sample pairs, Seo et al. cohort (23), and Riaz et al. cohort (25) were obtained from previous publication (16). The proliferation and immune infiltration scores were obtained from previous publication (26). The Tumor Immune Estimation Resource (TIMER) scores were downloaded from TIMER2.0 (http://timer.cistrome.org/) (27).

For the tumor-only analyses, we focused on BRCA, LGG, OV, LUAD, UCEC, HNSC, SKCM, KIRC, STAD, and LUSC, as these cancer types have >150 samples with all types of features available (mRNA expression, miRNA expression, APA estimation, immune and proliferation scores, and TIMER score) (**Supplementary Table 1**). For the paired tumor–normal analyses, we focused on BRCA, KIRC, HNSC, STAD, LUAD, and LUSC, as these cancer types have >5 sample pairs. For the survival analysis, we kept only BRCA, KIRC, and HNSC, as these cancers have a sample size of ≥40.

### Estimation of the number of miRNA target sites at transcriptome scale by PRIMATA-APA

To estimate the number of miRNA target sites at the transcriptome scale, we utilized our previously published bioinformatics tool, PRIMATA-APA, to calculate the numTS for each miRNA family. PRIMATA-APA takes mRNA abundance, estimated 3′UTR length dynamics, and annotated genome location of miRNA target site into consideration to infer the number of miRNA target sites by the following equations:


(1) $$miR_{PDUI\left(x,\;miR_{j}\right)}=\left(pUTR\left(x,miR_{j}\right)+dUTR\left(x,miR_{j}\right)*PDUI\left(x\right)\right)\;*FPKM\left(x\right)$$

(2) $$miR_{PDUI\left(miR_{j}\right)}=\sum_{x}miRs\left(x,miR_{j}\right)$$

In Equation 1, we estimated the number of target sites of *miR_j_* carried by the transcripts of gene *x*. To quantify this, we first assumed that transcript *x* has a constitutive proximal 3′UTR (pUTR) and a distal 3′UTR (dUTR). The genomic coordinate where pUTR ends and the *PDUI*(*x*) were estimated by one of the widely used tools, DaPars (18). Then, based on TargetScan prediction and the estimated end of pUTR, we defined $pUTR\left(x,miR_{j}\right)$ and $dUTR\left(x,miR_{j}\right)$ as the number of *miR_j_* target sites in pUTR and dUTR, respectively (15). Finally, the weighted abundance of *miR_j_* target sites on gene *x* was multiplied by the transcript abundance of gene *x*, *FPKM*(*x*). In Equation 2, we added up the number of *miR_j_* target sites carried by each gene *x* over all expressed genes to derive the global abundance of the target site of *miR_j_*, which was denoted as numTS in the paper. A more detailed description of the tool can be found in the method paper (19).

To make fair comparisons between miRNA numTS and expression, we matched numTS features and expression features by miRNA family in the run of PRIMATA-APA so that miRNA numTS and miRNA expression would have the same number of features in all the downstream analyses, including the correlation analysis, dimension reduction, and patient clustering analysis, and all cancer hallmark predictive models.

### Correlation analyses between miRNA numTS and expression

To test the hypothesis that the miRNA binding activity is disrupted in tumors, we first matched tumor samples and normal samples from the same patients. Then, we calculated the squared Spearman’s correlation coefficient between miRNA numTS and expression in tumor and normal samples separately. To assess the difference, we conducted a Wilcoxon rank-sum test on the squared correlation.

Due to the relatively small numbers of patients who have all types of measurements available in both tumor and matched normal samples, we enlarged the sample size of correlation analysis by focusing on only tumor samples. Similarly, Spearman’s correlation test was conducted to assess if a miRNA has significantly (Benjamini–Hochberg (BH)-corrected p< 0.05) correlated miRNA numTS and expression.

### MiRNA predictive model training and external validation

To comprehensively evaluate the potentials of miRNA numTS as a biomarker for cancer hallmarks, for each tumor sample, we obtained a series of signature scores from literature characterizing different aspects of cancer status, including immune and proliferation scores (26), and TIMER scores estimating the abundance of CD4 T cell, CD8 T cell, B cell, macrophage, neutrophil, and myeloid dendritic cell (27).

Then, with each of these scores as the outcome, miRNA numTS-based and miRNA expression-based predictive models were trained separately using elastic net regression conjugated with nested cross-validation. In the outer loop of the nested cross-validation, the whole dataset was split into a train set (75%) and a test set (15%). In the inner loop, the optimization was performed by 10-fold cross-validation on the train set where the optimal α (weight of L1 and L2 norm parameter, 10 possible values from 0 to 1, by 0.1) and β (penalty parameter) that lead to the most parsimonious model whose error is no more than one standard error above the lowest cross-validation error (1se) were selected. This procedure was repeated 300 times and root-mean-square error (RMSE) was calculated as the performance metric. The glmnet 4.1.4 R package was used to build the model (28).

To verify that the performance assessment results hold when models are trained by different statistical learning methods, three training methods widely adopted in the field were employed to replicate the performance assessment: random forest [rf from randomForest 4.7-1 R package (29)], support vector machine with polynomial kernel [svmPoly from kernel 0.9-30 R package (30)], and elastic net regression [glmnet from glmnet 4.1.4 R package (28) as control]. The assessment procedure was conducted using caret 6.0 R package (31).

To evaluate if the selected predictive miRNAs are generalizable, we conducted elastic net regression conjugated with bootstrapping to select stable sets of predictive miRNAs from two independent cohorts, TCGA LUAD and Seo et al. cohort (23). For each cohort, we first created 200 bootstrap samples from the original dataset by resampling with replacement. Then, with each bootstrap sample, we selected miRNAs using elastic net regression. We chose the optimal model by 10-fold cross-validation. This step would generate 200 sets of selected miRNAs from 200 bootstrap samples. Then, we calculated the frequency of being selected for each miRNA across 200 sets of selected miRNAs. The miRNAs with the top 50% frequency were considered predictive miRNAs for each cohort. Finally, we used a hypergeometric test to test if the predictive miRNAs from two cohorts were significantly overlapped.

### NumTS analysis on immunotherapy-treated melanoma cohort

To explore the potential of numTS features in distinguishing responders and non-responders to immunotherapies, we downloaded the RNA-Seq data of 105 advanced melanoma samples published by Riaz et al. (25). We conducted principal component analysis (PCA) dimension reduction on numTS features of miRNAs that were identified to be associated with the TIMER score of six cell types. We used the same elastic net regression conjugated with the bootstrapping approach (as described in the previous section) to identify such TIMER score-associated miRNAs on TCGA SKCM data. We used 70% frequency of being selected as the cutoff. The PCA with 95% confidence ellipses was drawn using stats 4.2.0 R package factoextra 1.0.7 R package (31). To quantify the separation of each pair of groups, we calculated the Mahalanobis distance between every two groups.

### Survival analyses

To assess the prognostic value of clinical features, we first used a Cox proportional-hazards model to associate survival time with clinical features, including age, gender (except for BRCA), and pathological stage (clinical-only model). Then, we estimated the change of numTS of the tumor *vs.* normal (ΔnumTS). To determine which miRNA ΔnumTS features contribute to the model, we fit a Cox model conjugated with LASSO feature selection using glmnet4.1.4 R package (28). In the feature selection, the clinical features were not penalized and always selected. We chose the optimal miRNAs by 10-fold cross-validation. Then, we added them to the clinical-only model and obtained the numTS-clinical model. To assess if the expression level of the selected miRNAs can also contribute to the model, we also added the expression level of the selected miRNAs into the clinical-only model and obtained the expr-clinical model.

Then, we sought to assess the performance of three models. Based on the predicted hazard estimation of each model, we classified patients into low- and high-risk groups, which were then visualized using the Kaplan–Meier plots and compared using the log-rank test. To further compare the three models, we used a likelihood ratio test (LRT) and compared the improvements made by numTS-clinical models and expr-clinical models on top of the clinical-only models.

## Results

### The correlation between miRNA expression and the number of putative miRNA target sites is disrupted in tumors

To examine numTS as an independent feature from miRNA expression, we first analyzed the correlation between numTS and miRNA expression in normal and tumor samples separately using four cancer types in TCGA, which have at least 20 tumor–normal sample pairs in both RNA-Seq and miRNA-Seq data (**Supplementary Table 1**) (see Materials and Methods). As miRNA binding can be viewed as a stoichiometry between the two features, the correlation can represent the miRNA binding homeostasis that helps maintain a stable level of mRNA and protein production in normal conditions. In all four cancer types, we found that strong correlations in normal samples are significantly disrupted in tumor samples (**Figure 1C**). This result suggests that normal miRNA binding regulation is disrupted in tumors, indicating miRNA numTS as a potentially independent feature from miRNA expression in cancer.

To further confirm numTS as an independent feature from miRNA expression, we estimated Spearman’s correlation coefficient between the features in the 10 TCGA cancer types that provide the RNA-Seq data for more than 180 tumor samples (**Supplementary Table 1**) (see Materials and Methods). In each of the cancer types, we divided the miRNAs into those whose expression level and numTS are correlated (BH-adjusted p-value<0.05) and not correlated in tumors. The results confirm that numTS is an independent measure of miRNA expression as follows. First, most miRNAs (on average 86.7%) do not have a significant (false discovery rate (FDR) p-value<0.05) correlation across the 10 cancer types (**Figure 1D**), consistent with the finding in **Figure 1B**. The number of miRNAs with a significant correlation is not related to the number of samples in each cancer type (**Supplementary Figures 1A, B**), suggesting that the lack of a significant correlation can be a biological signal, not due to the lack of power related to the small sample size. Second, the miRNAs with a significant correlation lowly overlap among three or more cancer types (**Figure 1E**), showing that these correlations are not meaningful to study biological mechanisms common to multiple cancer types. We will investigate their roles in cancer-specific mechanisms in the next section. Altogether, since the miRNA binding activity lost the homeostasis status between miRNA expression and numTS in tumors, the results suggest numTS as an independent molecular feature to study the miRNA binding activity in tumors.

### MiRNA numTS distinguishes cancer type-specific molecular mechanisms better than mRNA expression, miRNA expression, or APA degree

**Figure 2:**
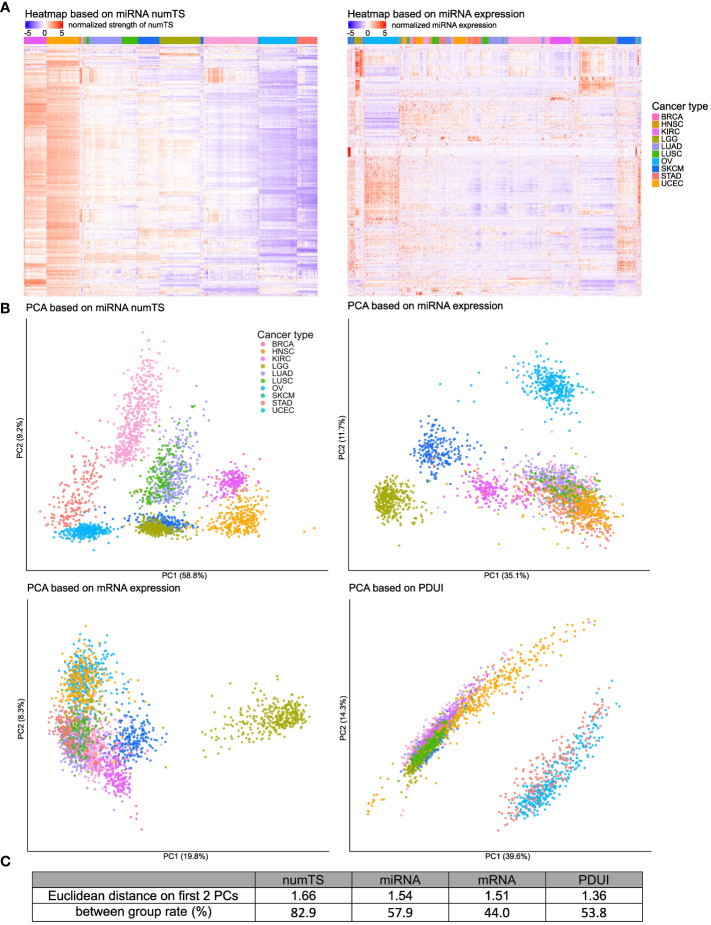
MiRNA numTS classifies cancer types better than other features. **(A)** Tumor samples from 10 cancer types were clustered based on miRNA numTS (left panel) or expression (right panel) using hierarchical clustering on Euclidean distance (see Materials and Methods). MiRNAs are in rows. Samples are in columns with the cancer type annotated on the top color bar. Values are log2 transformed, centered, and scaled by rows. **(B)** PCA dimension reduction based on miRNA numTS, miRNA expression, mRNA expression, and PDUI (see Materials and Methods). The distance metrics are shown in **(C)**. miRNA, microRNA; numTS, number of miRNA target sites; PCA, principal component analysis; PDUI, percentage of distal polyA site usage index.

To investigate how numTS represents cancer type-specific molecular mechanisms better than miRNA expression, we applied hierarchical clustering on the miRNA numTS or expression of 588 moderately expressed miRNAs from tumor samples across the 10 cancer types (see Materials and Methods). While the clustering using miRNA expression does not separate the samples by cancer type, miRNA numTS clearly groups the samples by cancer type (**Figure 2A**). This result demonstrates that miRNA numTS reflects the cancer type-specific miRNA-mediated mRNA regulation. Given that the miRNA expression generally varies more than numTS across tumor samples (**Supplementary Figure 2**), this result further supports that numTS holds more signal in a cancer type-specific manner and thus a more effective molecular feature than miRNA expression to study the cancer type-specific miRNA-mediated mRNA regulation.

To further show the strength of numTS to represent cancer type-specific biology, we compared numTS with other well-known molecular features: miRNA and mRNA expressions and PDUI of 12,717 expressed genes (see Materials and Methods). PDUI is an important molecular feature to estimate the degree of genes’ APA. Since APA is a major cause to change numTS across tumor samples, a comparison to the PDUI feature will indicate how specific numTS is to represent the putative miRNA binding affinity. Since the numbers of features in each feature type are different (304, 304, 12,717, and 225 for numTS, miRNA expression, mRNA expression, and PDUI, respectively), we preprocessed the data (see Materials and Methods) for a fair comparison. First, for each molecular feature, we projected samples to two principal component (PC) dimensions and annotated their cancer types in the figure (**Figure 2B**). Visual inspection of the PCA plot extends our observation from **Figure 2A**: the separation among cancer types is much clearer in the numTS features than miRNA expression, mRNA expression, or PDUI. To quantify the cancer-type separability using all features (not only PCs), we estimated the percentage of feature variance explained by cancer type (between-group rate) (**Figure 2C**). MiRNA numTS differentiates cancer types far better (82.9%) than all the other features (44%, 53.8%, and 57.9% in mRNA, PDUI, and miRNA expressions, respectively). Altogether, numTS is a novel molecular feature that differentiates cancer type-specific biology better than miRNA expression, mRNA expression, and PDUI.

### MiRNA numTS predicts tumor proliferation and cytotoxic immune infiltration status significantly better than miRNA expression in tumor microenvironment

**Figure 3:**
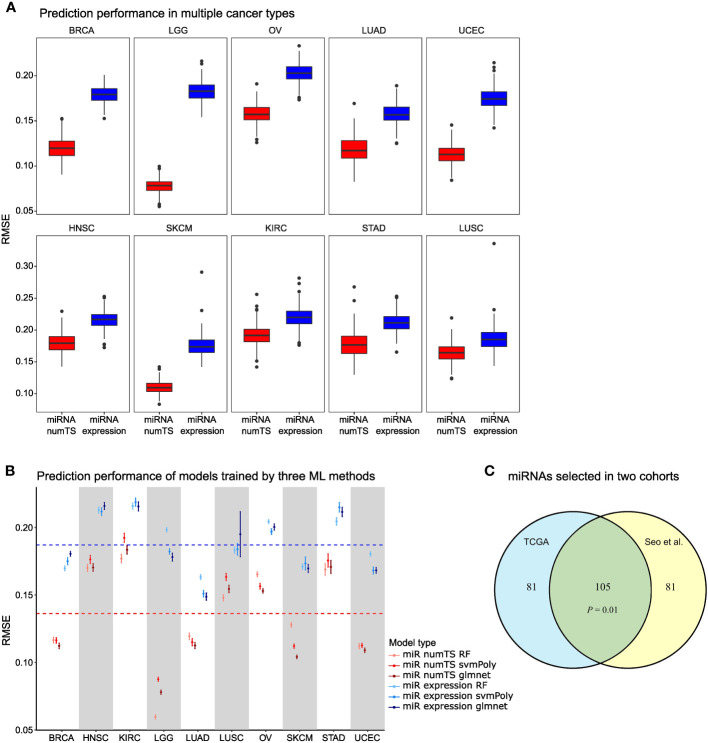
MiRNA numTS-based models outperform miRNA expression-based models in predicting tumor proliferation status. **(A)** Root-mean-square error (RMSE) as metric measuring the performance of numTS-based models and expression-based models predicting proliferation score. **(B)** RMSE of numTS-based models and expression-based models trained by three statistical learning methods (RF, random forest; svmPoly, support vector machine with polynomial kernel; glmnet, elastic net). **(C)** The overlap of miRNAs selected in TCGA LUAD cohort and Seo et al. LUAD cohort. Hypergeometric p-value measuring the significance of enrichment is indicated. miRNA, microRNA; numTS, number of miRNA target sites; TCGA, The Cancer Genome Atlas.

To compare the variations of important biomarkers explained by numTS *vs.* miRNA expression, we obtained two scores that indicated fundamental tumor-associated properties: tumor cell proliferation and immune cell infiltration. That is, tumor cell proliferation measures how quickly a cancer cell copies its DNA and divides into two cells, and immune cell infiltration measures the degree of immune cells infiltrating tumor sites. In particular, we downloaded the scores estimated for the tumor samples of the 10 cancer types based on the genes characterizing the biological processes (26). Then, with each score as the outcome, we built regression-based prediction models for each cancer type with either miRNA expression or numTS features as predictors (see Materials and Methods). NumTS predicts both the proliferation and immune score far better than miRNA expression in all cancer types (**Figure 3A**; **Supplementary Figure 3A**), on average 42.7% decrease for the proliferation and 5.14% decrease for the immune score in RMSE across the 10 cancer types. Since both proliferation and immune score successfully indicated fundamental processes in tumor formation, progression, and response to immunotherapies (26, 32–34), the results demonstrate a superb performance of the numTS feature to represent the important aspects of the tumor microenvironment. To verify that this outperformance holds true in models trained by different statistical learning methods, we employed three training methods widely adopted in the field to replicate the performance assessment: random forest, support vector machine with polynomial kernel, and elastic net regression (as control). All predictive models performed better with numTS features, regardless of the training method (**Figure 3B**; **Supplementary Figure 3B**). Altogether, the results indicate that the improvement in predictive accuracy is due to the outperformance of the numTS feature rather than a training method.To assess the reproducibility of the numTS features, we performed the following experiments. First, we identified 10 miRNAs that are commonly associated with immune infiltration scores in the 10 cancer types we investigated (intersection of the miRNAs selected for miRNA numTS across **Figure 3A** boxplots). We found extensive evidence from previous studies supporting their role in various cancer types (**Supplementary Table 2**). Second, we compared the miRNAs whose numTS value predicts the proliferation and immune infiltration scores between two independent lung adenocarcinoma datasets: TCGA LUAD (n = 326) and Seo et al. cohort (n = 83). Due to the relatively small sample size of the Seo et al. cohort, we conducted stability analysis to select the top 50% predictive miRNAs based on how frequently the miRNAs are selected across 200 bootstrap samples (see Materials and Methods). The comparison showed a significant overlap between the predictive miRNAs selected from two cohorts for both proliferation and immune infiltration scores. For proliferation score prediction, a significant number (105, 56.4%, p-value = 0.01, see Materials and Methods) of the selected miRNAs were identified commonly in the cohorts (**Figure 3C**). For immune infiltration score prediction, a similarly significant number (114, 59.5% on average, p-value = 0.001, see Materials and Methods) of the selected miRNAs were commonly identified (**Supplementary Figure 3C**). Between the 105 and 113 miRNAs predictive for proliferation and immune infiltration scores, respectively, we identified 26 miRNAs whose numTS are predictive for both immune and proliferation scores in TCGA LUAD cohort and the independent Seo et al. cohort. Among the 26 miRNAs, 18 (75%) miRNAs have been reported to be biomarkers for cancer hallmarks (**Supplementary Table 2**). This result suggests that the numTS feature explains the variability of two important hallmarks of cancer in an accurate and reproducible fashion for all cancer types, thereby promoting its potential as a novel biomarker for prospective studies.

To examine the biological implication of the numTS features selected above, we investigated the 26 miRNAs whose numTS strongly predict both proliferation and immune infiltration scores in TCGA lung cancer cohort and the Seo et al. cohort. Among the 26 miRNAs, 18 (75%) miRNAs have been reported to be biomarkers for cancer hallmarks, including proliferation, migration, invasion, and suppression, as well as DNA damage, cell apoptosis, immune system regulation, and long-term survival for diverse types of cancer (**Supplementary Table 2**). Among these miRNAs, miR-346 is of particular interest. In addition to being identified as a biomarker for prostate and liver cancers (35, 36), it has been found to compete with miRNA-138 for binding to 3′UTR of human telomerase reverse transcriptase (hTERT) mRNA (37). The binding of miR-346 promotes the translation of hTERT mRNAs, while the binding of the competitor miRNA suppresses hTERT translation. This highlights the importance of considering the miRNA target sites, as it reveals which miRNA is more likely to function and consequently predict the fate of the mRNA transcripts. Altogether, our results demonstrate that the numTS profile predicts tumor proliferation/immune infiltrating scores in a reproducible and biologically meaningful fashion.

### MiRNA numTS reveals cell type-specific miRNA targeting activities in immune cells in the tumor microenvironment

**Figure 4:**
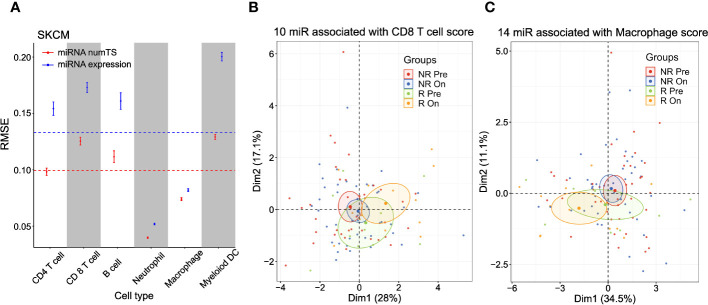
MiRNA numTS-based models outperform miRNA expression-based models in predicting tumor-infiltrating lymphocyte abundance. **(A)** Root-mean-square error (RMSE) as metric measuring the performance of numTS-based models and expression-based models predicting the abundance of tumor-infiltrating immune cells (TIMER scores of six cell types) of TCGA SKCM samples. The point in the middle of each vertical line represents the mean RMSE. The upper and lower bars represent mean ± 2SE (standard error). The horizontal dotted lines indicate the average RMSE across all cell types for miRNA numTS models and miRNA expression models separately. **(B, C)** PCA dimension reduction of immune checkpoint blockade-treated melanoma patients (Riaz et al. cohort). PCA plots were based on miRNAs that were associated with the abundance (estimated by TIMER score) of tumor-infiltrating CD8 T cells **(B)** and macrophages **(C)** in TCGA SKCM samples. The number of miRNAs selected for each cell type is indicated in the figure. miRNA, microRNA; numTS, number of miRNA target sites; TIMER, Tumor Immune Estimation Resource; TCGA, The Cancer Genome Atlas; PCA, principal component analysis.

Since the immune infiltration score is measured based on the genes characterizing cytotoxic effector immune cells (CD8+ T cells and NK cells), we further hypothesized that the numTS features may reflect the cell type-specific miRNA binding profiles expected from cell type-specific APA patterns (38). To test this hypothesis, we utilized a well-known immune cell abundance score in various cancer types, called TIMER score (27, 39), which was pre-calculated and made available for six immune cell types (B cell, macrophage, dendritic cell, neutrophil, CD4+ T cell, and CD8+ T cell) for TCGA data (see Materials and Methods). To predict the TIMER score for each immune cell type in each cancer type, we again built elastic net regression models conjugated with nested cross-validation (CV) using either miRNA numTS or expression as predictors. The numTS models predicted the TIMER score of six immune cells constantly better than miRNA expression in 59 comparisons (**Figure 4A**; **Supplementary Figures 4A, B**) except for one, CD4+ T cell in UCEC, where the miRNA numTS and expression models showed similar performance. In general, across all immune cell types and cancer types, the outperformance was shown in ranges from 0.7% (macrophage in LUSC) to 45.2% (neutrophil cell in LGG) in RMSE, with an overall 16% decrease. The results suggest that numTS reveals the miRNA binding profile specific to each cell type.

To demonstrate the role of the cell type-specific miRNA binding profile in immune regulation, we further analyzed ICB therapy trial data (25), where tumor-infiltrating immune cells play important roles in eliciting response. Since numTS features successfully predicted the miRNA binding profile of tumor-infiltrating immune cells, we examined if the numTS features can represent the treatment effect of immunotherapies. To answer this question, we selected the miRNA numTS features associated with the abundance of six immune cell types in TCGA skin cutaneous melanoma (SKCM) samples described in the previous paragraph. Then, we calculated the numTS values for the selected miRNAs using the RNA-Seq data of 105 advanced melanoma samples collected from patients before and after nivolumab (anti-PD-1 agent) and ipilimumab (anti-CTLA-4) administration (pre- and post-treatment, respectively) (25). PCA dimension reduction plots based on these numTS features clearly show a separation between post- and pre-treatment responder samples, while responder pre-treatment samples are located closer to non-responder samples of pre- and post-treatment (**Figures 4B, C**; **Supplementary Figure 4C**). For example, in the PCA plot with the macrophage, CD4 T cell, and myeloid dendritic cell-associated miRNA numTS features (**Figure 4C**; **Supplementary Figure 4C**), there is no overlap between 95% confidence ellipses of responder post- and non-responder groups (both pre- and post-treatment). Since responder post-treatment samples stand out from non-responder groups as well as responder pre-treatment samples, the result suggests a significant treatment effect of the ICB therapy in the numTS feature space. To further quantify this separation, we calculated the Mahalanobis distance between the sample groups (responders and non-responders, pre- or post-treatment, see Materials and Methods). In all the six cell types, responders (either pre- or post-treatment group) make the greatest overall distance to all the other groups (**Figures 4C**; **Supplementary Figure 4C**, **Supplementary Table 3**). Altogether, the results suggest the numTS features of miRNAs associated with tumor-infiltrating immune cells have potential to be a biomarker for patient’s response to immunotherapy.

### MiRNA numTS adds prognostic value beyond common clinical covariates and miRNA expression

**Figure 5:**
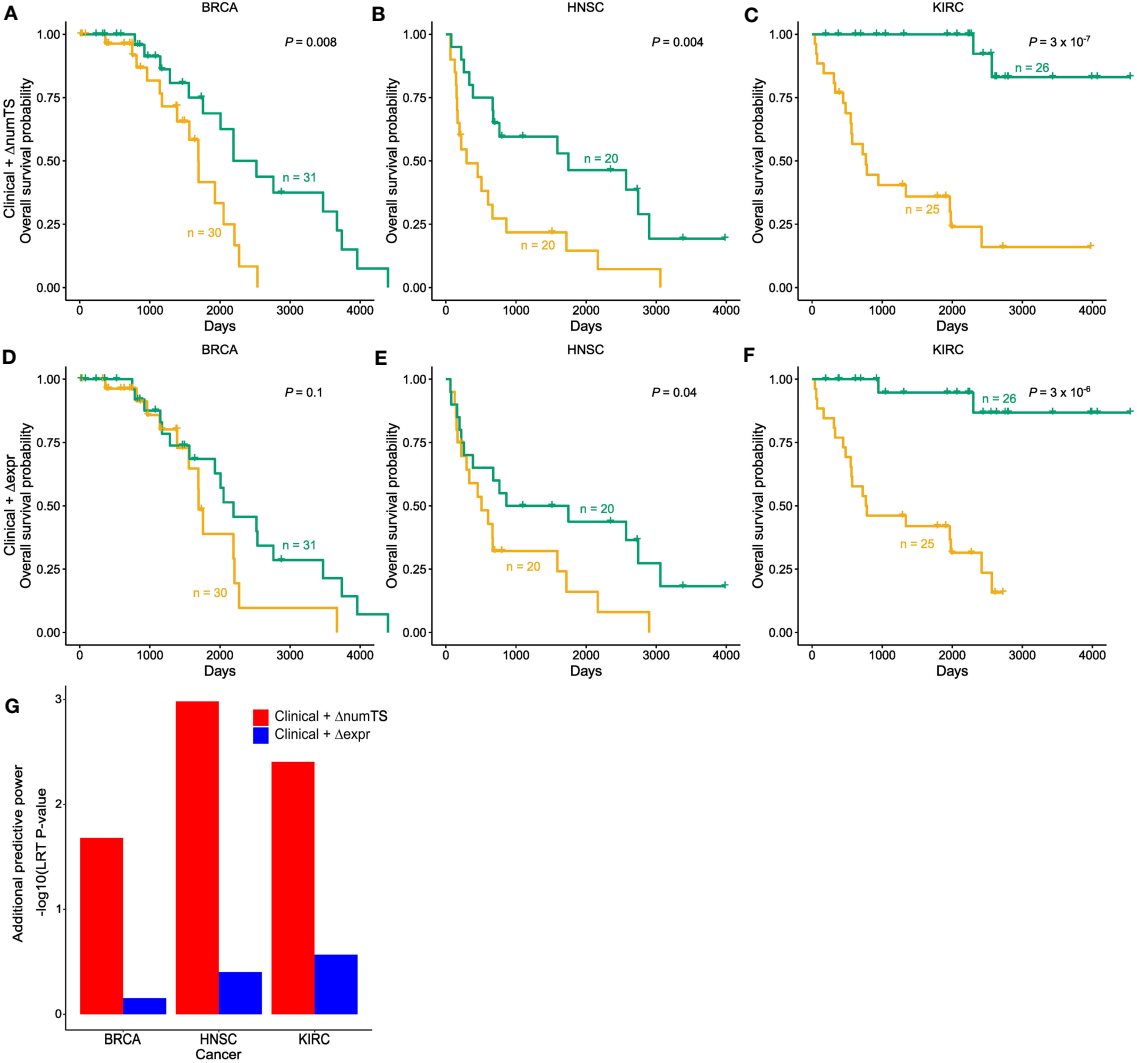
MiRNA ΔnumTS improves the survival model based on common clinical covariates by explaining additional variability. **(A–F)** Kaplan–Meier plots with high (yellow) and low (green) risk groups separated by **(A–C)** clinical features and ΔnumTS selected by LASSO. **(D–F)** Clinical features and Δexpression selected by LASSO. Sample sizes of high- and low-risk groups and log-rank test p-values are indicated in each figure. **(G)** Additional predictive power added by ΔnumTS and Δexpression, measured by likelihood ratio test comparing clinical + ΔnumTS model *vs.* clinical only model and clinical + Δexpression model *vs.* clinical only model. miRNA, microRNA; numTS, number of miRNA target sites; LASSO, least absolute shrinkage and selection operator.

To test whether numTS distinguishes patients with long-term survival from those with short-term survival, we compared models using clinical variables [including only tumor stage, age, gender (excluding breast cancer), and smoking status (lung cancer only)] plus the change of numTS in tumor *vs.* normal (numTS-clinical model) with models using the clinical covariates and the miRNA expressions (expr-clinical model) by building Cox proportional-hazards regression models (40). Here, we only used BRCA, HNSC, and KIRC, which provide sufficient sample size for RNA-Seq data (n ≥ 30, **Supplementary Table 1**). In all the cancer types, the numTS-clinical models significantly differentiate long-term survivors from short-term survivors, while clinical-only models do not (**Figures 5A–C**; **Supplementary Figures 5A–C**). The numTS-clinical model differentiates the two groups more significantly than expr-clinical models in all three cancer types (**Figures 5D–F**), suggesting that miRNA numTS explained additional variance of survival time that was not captured by miRNA expression. To quantify the added prognostic value of ΔnumTS compared to miRNA expression, we used an LRT and compared the improvements made by numTS-clinical models and expr-clinical models on top of the clinical-only models. The LRT results (**Figure 5G**) clearly demonstrate a strong and consistent additional prognostic power of numTS compared to miRNA expression. Together, in addition to commonly used clinical features, miRNA numTS provides better prognostic value for survival analysis than miRNA expression.

## Discussion

In this manuscript, we developed the statistical method BIOMATA-APA to find that numTS is an effective biomarker faithfully representing miRNA binding mechanisms. In particular, by running BIOMATA-APA on TCGA and independent cohort data both with immunotherapy and no immunotherapy, we demonstrated for the first time that the numTS feature 1) distinguishes different cancer types, 2) predicts tumor proliferation and immune infiltration status, 3) explains variation in the proportion of tumor-infiltrating immune cells, 4) predicts response to ICB therapy, and 5) adds prognostic power beyond clinical and miRNA expression. Notably, the results on the different cancer types and proliferation and immune infiltration status suggest that different cancer types may use the miRNA binding mechanism differently in relation to the unique APA landscape, and the miRNA binding mechanism is also involved in the interactions between tumor proliferation and immune infiltration in the tumor microenvironment. Also, the results on the ICB therapy response and the prognostic power reveal that the miRNA binding mechanisms play important roles in eliciting immunotherapy responses in cancer patients. Based on this, our work with BIOMATA-APA elucidates a novel layer of intricate mechanisms where widespread and cancer type-specific APA events extensively interact with the miRNA regulatory axis.

Our work on numTS and with BIOMATA-APA is novel compared to previous miRNA biomarker identification work. Previously, multiple miRNAs have been demonstrated to have regulatory roles in cancers. However, the miRNA expression-based biomarkers for cancer hallmarks or clinical outcomes have not been widely used due to their relatively poor reproducibility. This work suggests that the poor reproducibility may be attributable to the fact that 1) miRNAs function by binding to the target sites located on mRNA transcripts (18), 2) the target sites are globally disrupted in tumor samples (18), and 3) the target sites have not been considered as a biomarker. To fill this knowledge gap, we suggest identifying reproducible miRNA biomarkers by considering the miRNA target sites (numTS). In fact, for two cancer hallmark scores, tumor cell proliferation and immune infiltration scores, we replicated 56.4% and 59.5% of miRNA numTS biomarkers that we found in TCGA data in an independent cohort, respectively. Also, miRNA numTS can be more widely usable than miRNA expression since the estimation of numTS does not require miRNA expression data and only requires regular RNA-Seq data. It is a great advantage of the numTS feature because researchers can conduct miRNA research even if they do not have miRNA expression.

To fully examine the dynamic miRNA binding activity, the following studies are needed. First, BIOMATA-APA should additionally incorporate not only miRNA expression but also other aspects of the RNA-level regulations. For example, long non-coding RNAs (lncRNAs (41)) and RNA-binding proteins (RBPs (42)) are known to affect miRNA target site dynamics as “sponges” of miRNAs or be involved in miRNA processing pathways, respectively. Since both lncRNAs and RBPs play instrumental roles in cancer progression and regulation, understanding how these molecules influence physiological or clinical outcomes through their global target site dynamics can provide novel insights into cancer research (43). Second, modeling the miRNA binding dynamics should eventually be at the single-cell level. Once BIOMATA-APA is extended to model miRNA binding dynamics at the single-cell level, it can further be combined with other single cell-level models that can identify co-expressed gene modules (44) or cell–cell communication [ligand–receptor interactions (45)] to elucidate comprehensive single-cell molecular processes. Notably, since the method for cell–cell communication considers a novel adaptive graph model with attention mechanisms for interacting mRNAs, it would be interesting to extend the attention mechanisms to incorporate the mRNA–miRNA binding interactions. Third, while this manuscript demonstrated the promise of predicting immunotherapy response using the miRNA numTS features identified in TCGA data, miRNA numTS features further need to be identified from the patients who underwent the therapies, not from TCGA patients. Although currently, we cannot use the patient data of immunotherapy for numTS identification due to the limited number of samples from responders (10 pre-treatment samples and 13 post-treatment samples), we will seek to cross-validate our observations in a larger cohort in the future.

By providing a comprehensive understanding of the miRNA binding activity, BIOMATA-APA allows us to elucidate the biological functions and potential therapeutic applications of miRNAs. Notably, now that mRNA vaccines, like the Pfizer-BioNTech (46) and Moderna (47) COVID-19 vaccines, have gained significant attention and success, BIOMATA-APA will potentially improve both the efficacy and safety of such mRNA vaccines by fine-tuning their interactions with miRNAs. Several studies demonstrated that the immune-related genes changed their interaction with miRNAs (19, 38) and that the interactions between immune-related genes and miRNAs play an important role in COVID-19 disease (48, 49). Thus, BIOMATA-APA can potentially fine-tune the immune response to achieve the desired effect. For example, as mRNA and miRNA profiles vary among individuals, the vaccine’s effectiveness can be optimized by avoiding or promoting the interaction of specific mRNAs and miRNAs given the individual’s numTS profile. Also, since multiple mRNAs would interact with multiple miRNAs, a comprehensive understanding of miRNA interactions with mRNAs can help avoid unintended interactions, which can help improve the vaccine’s safety profile.

To the best of our knowledge, this is the first pan-cancer study to systematically demonstrate numTS as a novel type of biomarker representing the miRNA binding effects underlying tumorigenesis and pave the way to incorporate miRNA target sites for miRNA biomarker identification.

## Data availability statement

The original contributions presented in the study are included in the article/**Supplementary Material**. Further inquiries can be directed to the corresponding authors.

## Ethics statement

Ethical approval was not required for the studies involving humans because we used only publicly available data sets. The studies were conducted in accordance with the local legislation and institutional requirements. Written informed consent for participation was not required from the participants or the participants’ legal guardians/next of kin in accordance with the national legislation and institutional requirements because we used only publicly available data sets. Ethical approval was not required for the study involving animals in accordance with the local legislation and institutional requirements because we used only publicly available data sets.

## Author contributions

YB implemented the software, ran the experiments, and interpreted the results. YL ran the experiments. YQ interpreted the results. XY implemented the software. GT implemented the software. SK interpreted the results and wrote the manuscript. HP interpreted the results, wrote the manuscript, and conceptualized this project. All authors contributed to the article and approved the submitted version.
