# Supplementary material for: The microRNA target site profile is a novel biomarker in the immunotherapy response
Source: Front Oncol. 2023 Dec 21;13:1225221. doi: 10.3389/fonc.2023.1225221 (PMC10771317; doi:10.3389/fonc.2023.1225221)
Supplement: Supplementary file 1 [file DataSheet_1.pdf]

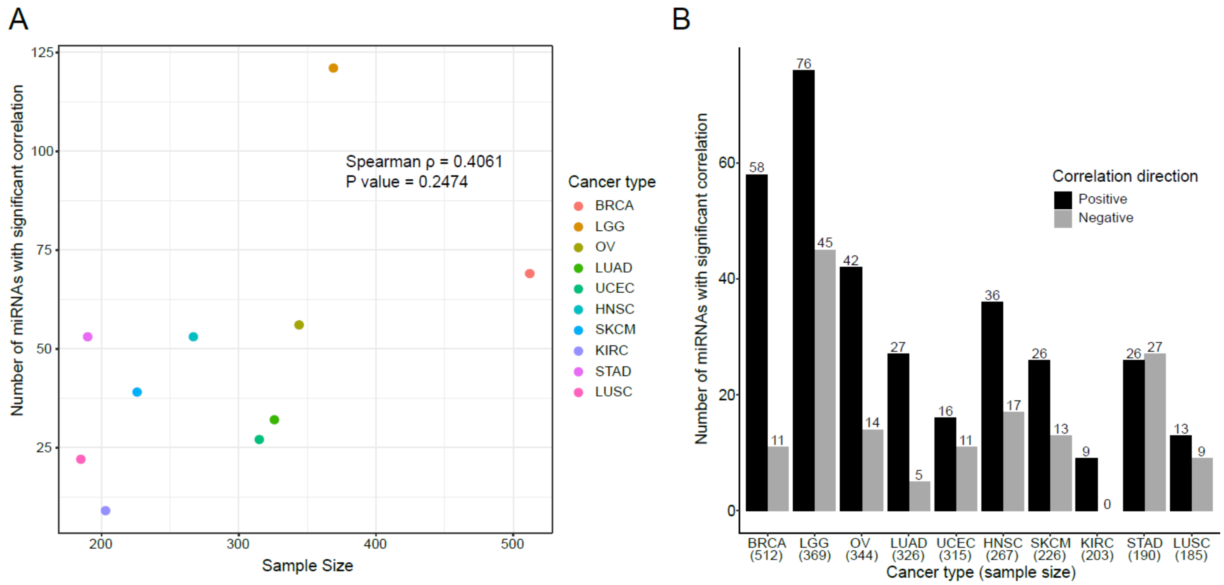

Supp. Fig. 1 | Number of miRNA target sites (numTS) is a novel biomarker independent from miRNA expression. A, The number of miRNAs with significant correlation between numTS and expression is not related to the sample size of each cancer type. **B**, Among the miRNAs with significant correlation between miRNA expression and numTS, there is no significant trend to either direction.

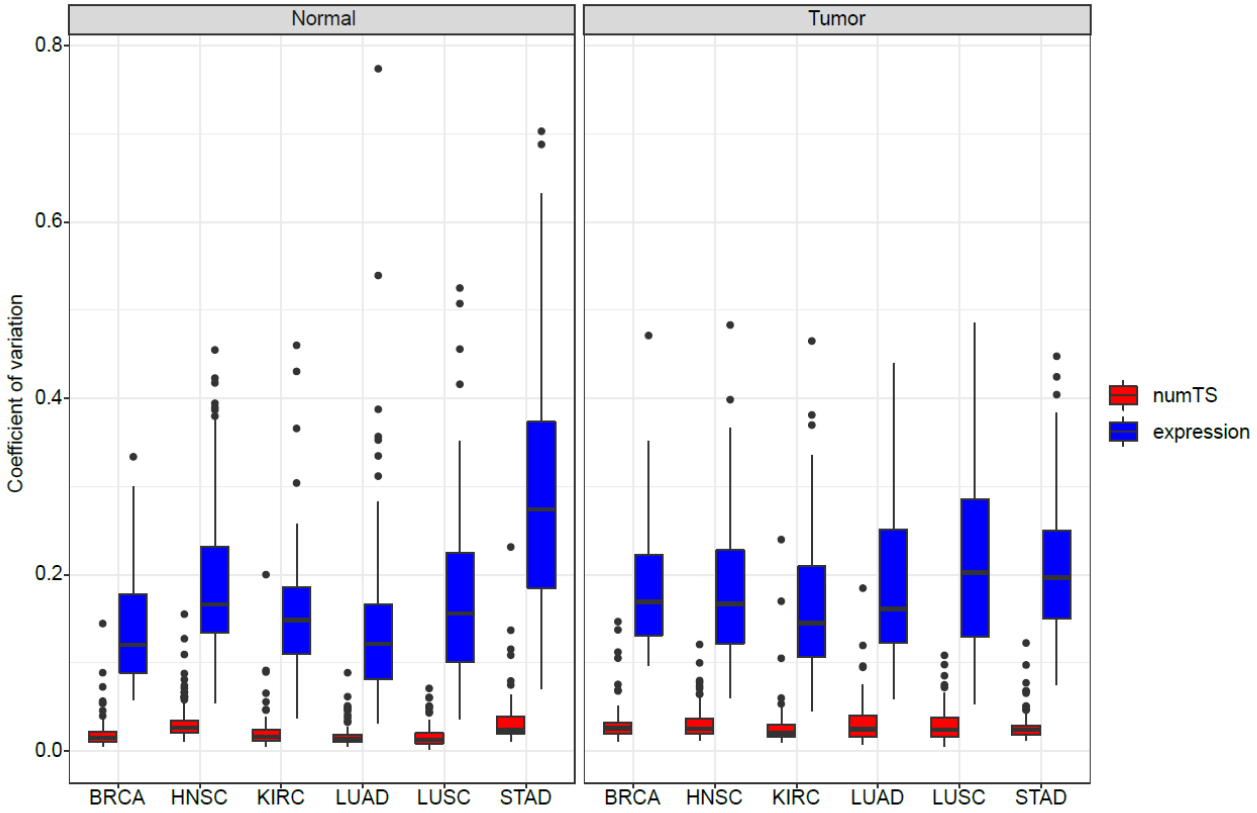

Supp. Fig. 2 | MiRNA expression varies more than miRNA numTS in all cancer types. The coefficient of variation of miRNA expression and numTS in each cancer type.

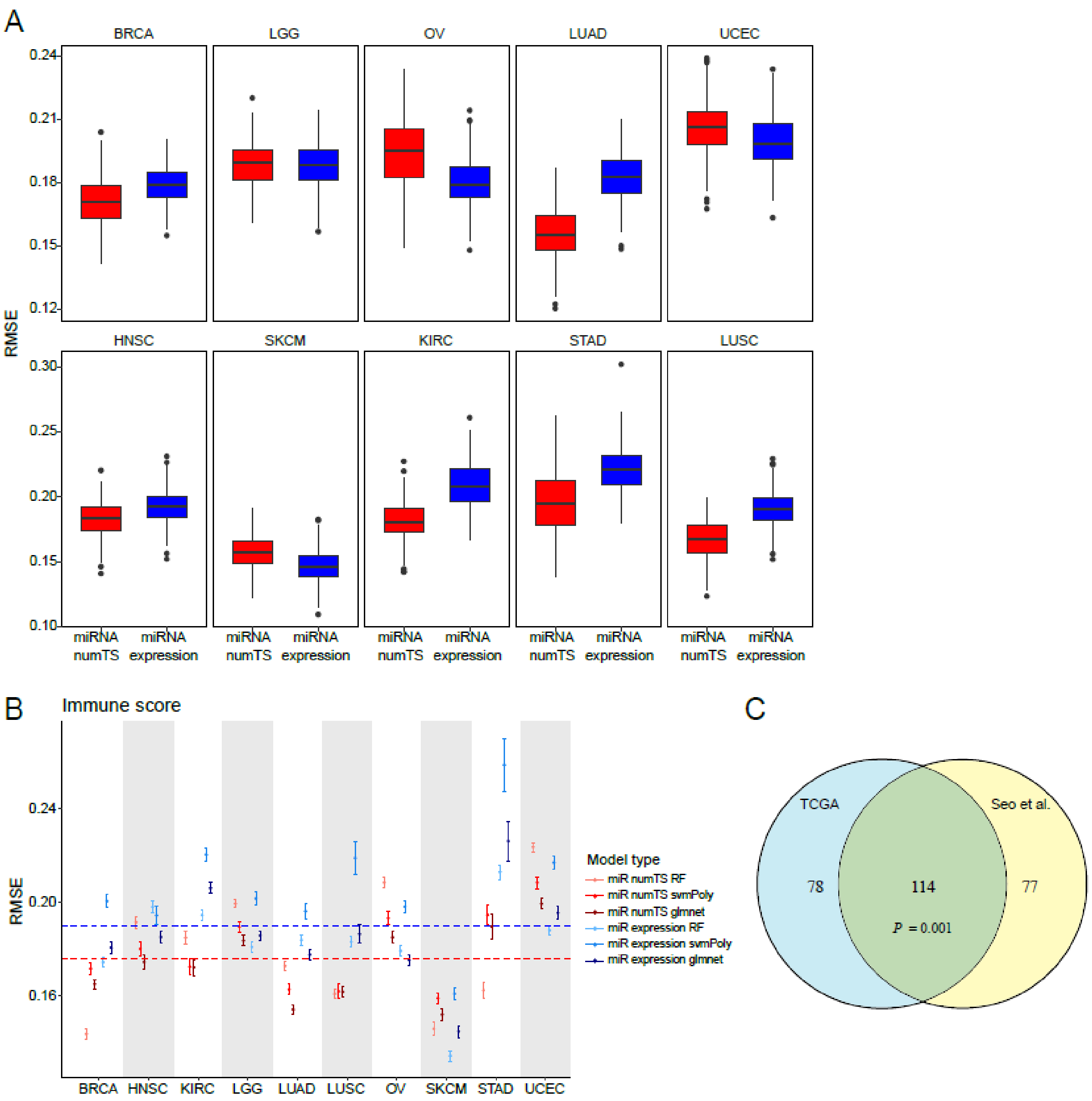

Supp. Fig. 3 | MiRNA numTS-based models outperform miRNA expression-based models in predicting tumor immune status. A, Root-mean-square error (RMSE) as metric assessing the performance of numTS-based model and expression-based model predicting immune score by nested 10-fold cross-validation. **B,** RMSE of numTS-based models and expression-based models trained by three statistical learning methods (RF: random forest; svmPoly: support vector machine with polynomial kernel; glmnet: elastic net). **C**, The overlap of miRNAs selected in TCGA LUAD cohort and Seo et al. LUAD cohort. Hypergeometric P value measuring the significance of enrichment is indicated.

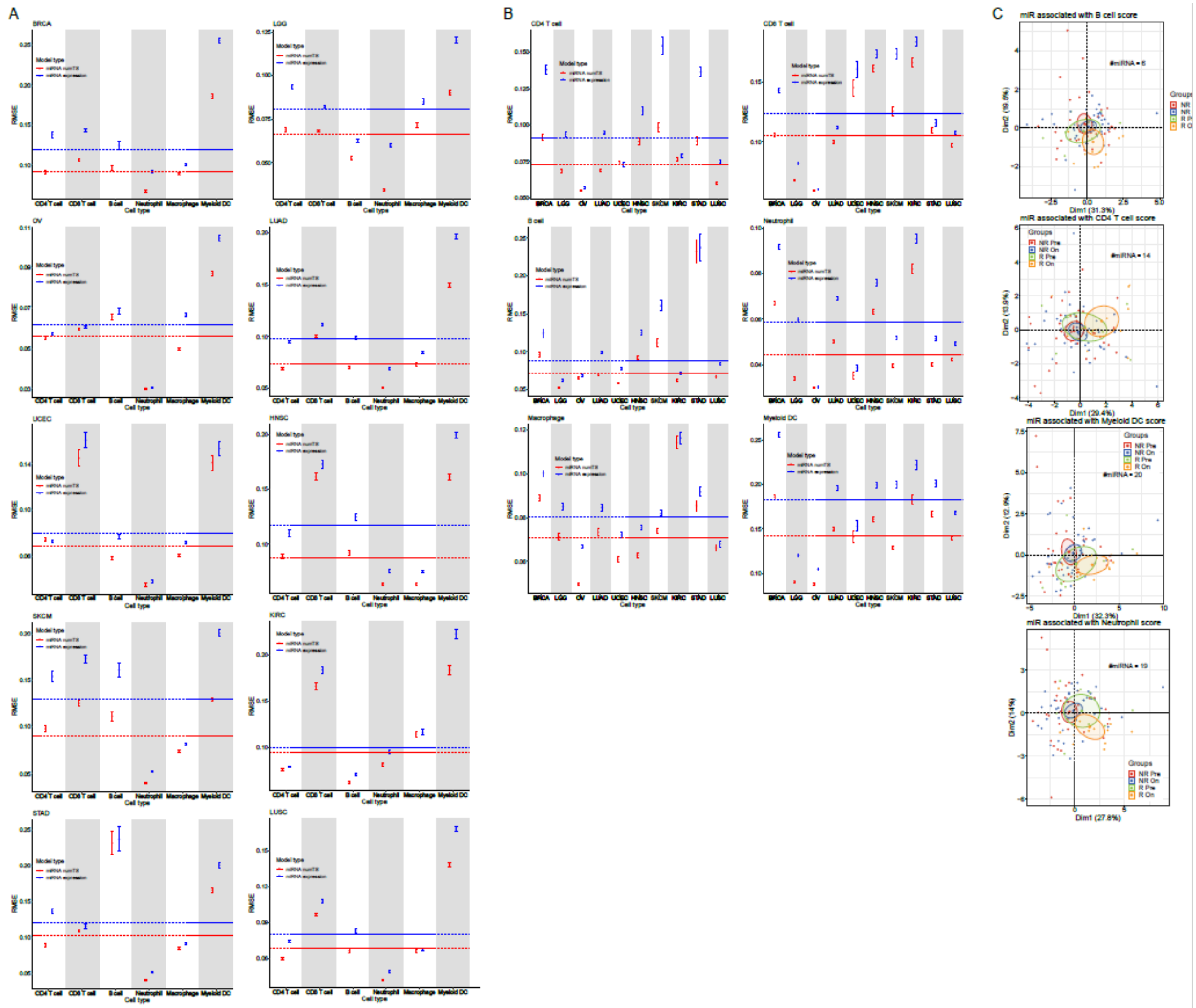

Supp. Fig. 4 | MiRNA numTS-based models outperform miRNA expression-based models in predicting tumor infiltrating lymphocytes abundance and show potential in reflecting response to immunotherapies. A and B, Root-mean-square error (RMSE) as metric measuring the performance of numTS-based models and expression-based models predicting TIMER scores of six cell types. Figure A is organized by cancer type and Figure B is organized by cell type. The point in the middle of each vertical line represents the mean RMSE. The upper and lower bars represent mean ± 2SE (standard error). The horizontal dotted lines indicate the average RMSE across all cell types for miRNA numTS models and miRNA expression models separately. **C,** PCA dimension reduction of immune-checkpoint blockade treated melanoma patients (Riaz et al. cohort). PCA plots were based on miRNAs that were associated with the abundance (estimated by TIMER score) of tumor infiltrating immune cells (B cell, CD4 T cell, Myeloid dendritic cell, Neutrophil) in TCGA SKCM samples. The number of miRNA selected for each cell type was indicated in the figure.

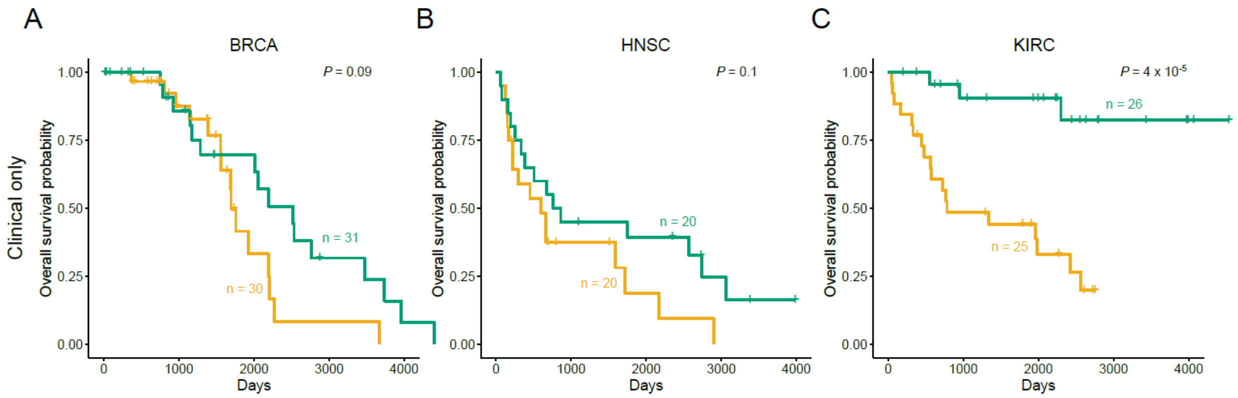

Supp. Fig. 5 | MiRNA ΔnumTS improves the survival model based on common clinical covariates by explaining additional variability. A-C, Kaplan-Meier plots with high (yellow) and low (green) risks groups separated by clinical features only.
