# Supplementary material for: The microRNA target site profile is a novel biomarker in the immunotherapy response
Source: Front Oncol. 2023 Dec 21;13:1225221. doi: 10.3389/fonc.2023.1225221 (PMC10771317; doi:10.3389/fonc.2023.1225221)
Supplement: Supplementary file 2 [file Table_1.docx]

**Supplementary table 1, Data description**

| Sample used | Data Source | | | Cancer type | Number of samples with all data available (for tumor only analyses: mRNA expr., miR expr., PDUI, immune&proliferation score ***; for tumor normal pair analyses: mRNA expr., miR expr., PDUI, survival info; for validation ). |
| --- | --- | --- | --- | --- | --- |
|  | mRNA expression * | miRNA expression ** | PDUI |  |  |
| Tumor only | TCGA | TCGA | TC3A | BRCA | 512 |
| Tumor only | TCGA | TCGA | TC3A | LGG | 369 |
| Tumor only | TCGA | TCGA | TC3A | OV | 344 |
| Tumor only | TCGA | TCGA | TC3A | LUAD | 326 |
| Tumor only | TCGA | TCGA | TC3A | UCEC | 315 |
| Tumor only | TCGA | TCGA | TC3A | HNSC | 267 |
| Tumor only | TCGA | TCGA | TC3A | SKCM | 226 |
| Tumor only | TCGA | TCGA | TC3A | KIRC | 203 |
| Tumor only | TCGA | TCGA | TC3A | STAD | 190 |
| Tumor only | TCGA | TCGA | TC3A | LUSC | 185 |
| Tumor normal pair | TCGA | TCGA | Xiang et al. | BRCA | 61 |
| Tumor normal pair | TCGA | TCGA | Xiang et al. | KIRC | 51 |
| Tumor normal pair | TCGA | TCGA | Xiang et al. | HNSC | 40 |
| Tumor only | Seo et al. | NA | Xiang et al. | LUAD | 83 (no miR expression) |
| Tumor only | Riaz et al. | NA | Wang et al. | melanoma | 105 (no miR expression) |

*The threshold for mRNA expression is mRNA expression average across all samples greater than 1 FPKM and smaller than 100 FPKM.

**The threshold for miR expression is miR family expression average across all samples greater than 0.01 FPM.

***Immune and proliferation score were downloaded from Davoli et al.
