# Supplementary material for: The microRNA target site profile is a novel biomarker in the immunotherapy response
Source: Front Oncol. 2023 Dec 21;13:1225221. doi: 10.3389/fonc.2023.1225221 (PMC10771317; doi:10.3389/fonc.2023.1225221)
Supplement: Supplementary file 3 [file Table_2.docx]

**Supplementary table 2, Publications supporting the biological function of the selected miRNAs**

| miRNAs | Publications |
| --- | --- |
| miR-346 | miR-346 and miR-138 competitively regulate hTERT in GRSF1- and AGO2-dependent manners, respectively |
|  | miRNA‑346 promotes proliferation, migration and invasion in liver cancer |
|  | A non-coding RNA balancing act: miR-346-induced DNA damage is limited by the long non-coding RNA NORAD in prostate cancer |
| miR-1193 | MiR-1193 Inhibits the Malignancy of Cervical Cancer Cells by Targeting Claudin 7 (CLDN7) |
|  | Inhibition of miR-1193 leads to synthetic lethality in glioblastoma multiforme cells deficient of DNA-PKcs |
| miR-581 | MiR-581/SMAD7 Axis Contributes to Colorectal Cancer Metastasis: A Bioinformatic and Experimental Validation-Based Study |
| miR-300 | miR‑300 regulates tumor proliferation and metastasis by targeting lymphoid enhancer‑binding factor 1 in hepatocellular carcinoma |
|  | miR-300 inhibits epithelial to mesenchymal transition and metastasis by targeting Twist in human epithelial cancer |
|  | MicroRNA‑300 inhibits the growth of hepatocellular carcinoma cells by downregulating CREPT/Wnt/β‑catenin signaling |
| miR-544b | miR-544 promotes maturity and antioxidation of stem cell-derived endothelial like cells by regulating the YY1/TET2 signaling axis |
| miR-665 | MicroRNA-665 facilitates cell proliferation and represses apoptosis through modulating Wnt5a/β-Catenin and Caspase-3 signaling pathways by targeting TRIM8 in LUSC |
|  | miR-665 expression predicts poor survival and promotes tumor metastasis by targeting NR4A3 in breast cancer |
|  | MicroRNA-665 Regulates Cell Proliferation and Apoptosis of Vascular Smooth Muscle Cells by Targeting TGFBR1 |
| miR-3918 | MiR-3918 Inhibits Tumorigenesis of Glioma via Targeting EGFR to Regulate PI3K/AKT and ERK Pathways |
| miR-3178 | miR-3178 inhibits cell proliferation and metastasis by targeting Notch1 in triple-negative breast cancer |
|  | miR-3178 as a prognostic indicator and tumor suppressor of gastric cancer |
| miR-614 | Upregulation of miR-614 promotes proliferation and inhibits apoptosis in ovarian cancer by suppressing PPP2R2A expression |
|  | miR614 Expression Enhances Breast Cancer Cell Motility |
| miR-611 | miR-611 promotes the proliferation, migration and invasion of tongue squamous cell carcinoma cells by targeting FOXN3 |
| miR-3131 | The Relationship between Pre-miR-3131 3-bp Insertion/Deletion Polymorphism and Susceptibility and Clinicopathological Characteristics of Patients with Breast Cancer |
| miR-3687 | Upregulation of miR-3195, miR-3687 and miR-4417 is associated with castration-resistant prostate cancer |
|  | miR-3687 Overexpression Promotes Bladder Cancer Cell Growth by Inhibiting the Negative Effect of FOXP1 on Cyclin E2 Transcription |
|  | High miR-3687 Expression Affects Migratory and Invasive Ability of Oesophageal Carcinoma |
| miR-612 | miR-612 suppresses the invasive-metastatic cascade in hepatocellular carcinoma |
|  | MiR-612 regulates invadopodia of hepatocellular carcinoma by HADHA-mediated lipid reprogramming |
| miR-3187-5p | Circulating serum exosomal miR-20b-5p and miR-3187-5p as efficient diagnostic biomarkers for early-stage non-small cell lung cancer |
| miR-1266 | MiR-1266 suppresses the growth and metastasis of prostate cancer via targeting PRMT5 |
|  | miR-1266 Contributes to Pancreatic Cancer Progression and Chemoresistance by the STAT3 and NF-κB Signaling Pathways |
|  | High expression of microRNA‑1266 in hepatocellular carcinoma is associated with poor prognosis of patients and biological cell growth |
|  | MiR-1266 promotes cell proliferation, migration and invasion in cervical cancer by targeting DAB2IP |
| miR-639 | miR-639 is associated with advanced cancer stages and promotes proliferation and migration of nasopharyngeal carcinoma |
|  | miR-639 regulates transforming growth factor beta-induced epithelial-mesenchymal transition in human tongue cancer cells by targeting FOXC1 |
|  | miR-639 promotes the proliferation and invasion of breast cancer cell in vitro |
| miR-3195 | Expressions of miR-122a and miR-3195 in laryngeal cancer and their effects on the proliferation and apoptosis of laryngeal cancer cell Hep-2 |
|  | Upregulation of miR-3195, miR-3687 and miR-4417 is associated with castration-resistant prostate cancer |
| miR-1294 | MicroRNA-1294 inhibits the proliferation and enhances the chemosensitivity of glioma to temozolomide via the direct targeting of TPX2 |
|  | Endosomal TLR-8 Senses microRNA-1294 Resulting in the Production of NFḱB Dependent Cytokines |
|  | Down-Regulation of MiR-1294 is Related to Dismal Prognosis of Patients with Esophageal Squamous Cell Carcinoma through Elevating C-MYC Expression |
