# Supplementary material for: The microRNA target site profile is a novel biomarker in the immunotherapy response
Source: Front Oncol. 2023 Dec 21;13:1225221. doi: 10.3389/fonc.2023.1225221 (PMC10771317; doi:10.3389/fonc.2023.1225221)
Supplement: Supplementary file 4 [file Table_3.docx]

**Supplementary table 3, Mahalanobis distance between each pair of groups**

| CD4 T cell | | | | |
| --- | --- | --- | --- | --- |
|  | R Pre | R On | NR Pre | NR On |
| R Pre | 0 |  |  |  |
| R On | 0.67 | 0 |  |  |
| NR Pre | 0.68 | 1.19 | 0 |  |
| NR On | 0.75 | 1.15 | 0.50 | 0 |
| CD8 T cell | | | | |
|  | R Pre | R On | NR Pre | NR On |
| R Pre | 0 |  |  |  |
| R On | 1.01 | 0 |  |  |
| NR Pre | 0.64 | 1.45 | 0 |  |
| NR On | 0.70 | 1.29 | 0.58 | 0 |
| B cell | | | | |
|  | R Pre | R On | NR Pre | NR On |
| R Pre | 0 |  |  |  |
| R On | 0.76 | 0 |  |  |
| NR Pre | 0.35 | 0.67 | 0 |  |
| NR On | 0.31 | 0.51 | 0.46 | 0 |
| Neutrophil | | | | |
|  | R Pre | R On | NR Pre | NR On |
| R Pre | 0 |  |  |  |
| R On | 0.88 | 0 |  |  |
| NR Pre | 0.79 | 1.16 | 0 |  |
| NR On | 0.80 | 0.97 | 0.53 | 0 |
| Macrophage | | | | |
|  | R Pre | R On | NR Pre | NR On |
| R Pre | 0 |  |  |  |
| R On | 1.01 | 0 |  |  |
| NR Pre | 1.25 | 1.09 | 0 |  |
| NR On | 1.34 | 1.08 | 0.38 | 0 |
| Myeloid DC | | | | |
|  | R Pre | R On | NR Pre | NR On |
| R Pre | 0 |  |  |  |
| R On | 1.01 | 0 |  |  |
| NR Pre | 1.25 | 1.09 | 0 |  |
| NR On | 1.34 | 1.08 | 0.38 | 0 |
